# An Extremely Rare Bile Leakage: Aberrant Bile Duct in Left Triangular Ligament (Appendix Fibrosa Hepatis)

**DOI:** 10.1155/2018/1259561

**Published:** 2018-01-31

**Authors:** İhsan Yıldız, Yavuz Savaş Koca, Sezayi Kantar

**Affiliations:** Department of General Surgery, Suleyman Demirel University Medical School, Isparta, Turkey

## Abstract

**Background:**

The anatomical variability of bile ducts can leave surgeons in very difficult conditions.Ultrasonography, computed tomography, magnetic resonance imaging (MRCP) and endoscopic imaging methods are used in diagnosis. In addition to conservative approaches, endoscopic procedures and laparoscopic or open surgical interventions may be necessary for treatment. In this article, we present a case of aberrant bile duct in left triangular ligament (appendix fibrosa hepatis), which is rarely seen.

**Case:**

We report the case of a 67-year-old female patient who was operated on due to dumping syndrome symptoms and hiatal hernia. There was a drainage of bile from the left side of the liver which was placed under the cardioesophageal junction. MRCP found bile esophageal in the left triangular ligament of the liver. Aberrant bile ducts were found in the left triangular ligament and ligated. The patient was discharged on the 7th day after operation.

**Conclusion:**

The anatomical variability of bile ducts can leave surgeons in very difficult conditions. We recommend that the dissected left triangular ligament should be ligated for the aberrant bile duct, especially in female patient.

## 1. Introduction

The structural anatomy of the bile ducts is highly variable and can be said to have almost no stable anatomy. This variability is more common in women [1]. This situation can often leave the surgeon in difficult situations during surgery on the liver, bile ducts, pancreas, and upper gastrointestinal tract [2].

Therefore, the structure of the bifurcation, especially the anatomy of the biliary tract, should be assessed very well before the operation, and possible ultrasonography, computed tomography, magnetic resonance cholangiopancreatography (MRCP), and endoscopic imaging methods should be used carefully [3, 4]. Nevertheless, after all, there may be bile leakage. Although the gallbladder surgery is performed commonly, bile leakage is seen after upper gastrointestinal system surgery for various reasons [5]. Endoscopic procedures and laparoscopic or open surgical interventions may be necessary, as well as conservative approaches such as waiting for spontaneous abortion of the leakage according to the patient's situation [3, 5, 6].

This patient was presented due to the fact that bile leakage was very extreme and there was only one case in the literature.

## 2. Case Presentation

**Figure 1:**
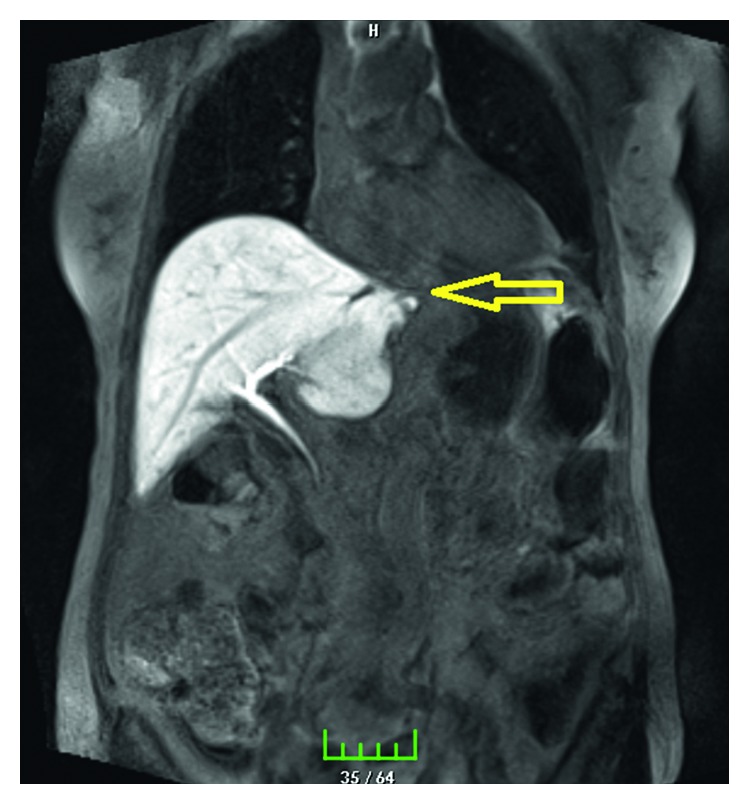
MRCP imaging. Bile leakage is shown in the left triangular ligament (appendix fibrosa hepatis).

**Figure 2:**
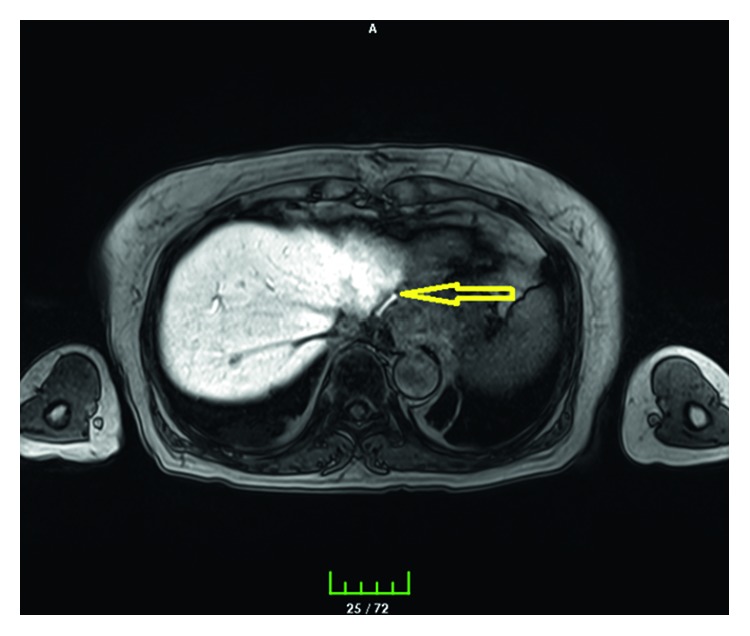
MRCP imaging. Bile leakage is shown in the drain.

**Figure 3:**
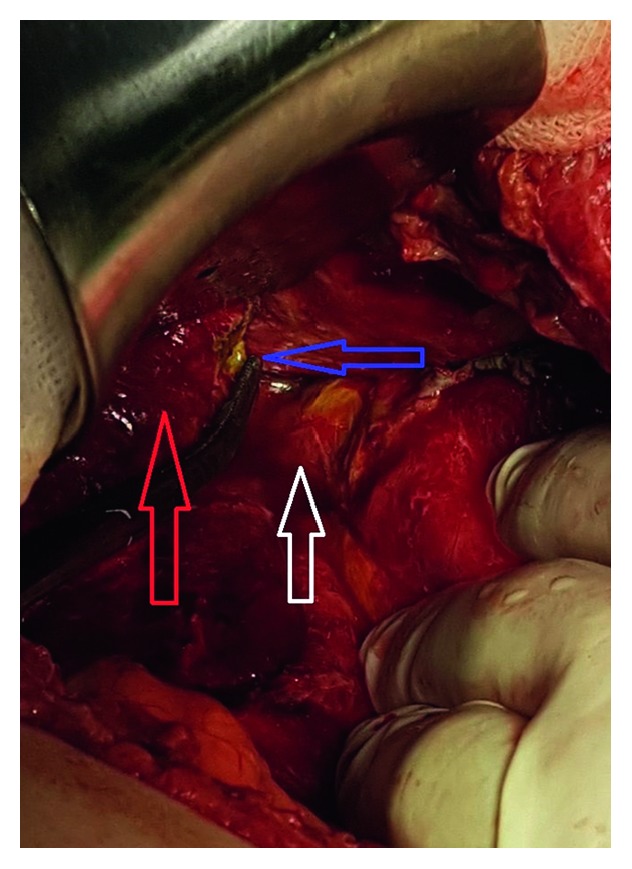
Left liver triangular ligament. Blue arrow shows bile leakage, red arrow shows the left triangular ligament, and white arrow shows the cardioesophageal junction.

A 67-year-old female underwent bilateral truncal vagotomy, cholecystectomy, Billroth-2 gastrectomy, and Roux-en-Y procedure for pyloric stenosis twenty years ago due to early dumping syndrome and sliding-type hiatal hernia. The liver left lobe was mobilized by dissecting the left triangular ligament (appendix fibrosa hepatis) to release the cardioesophageal junction and stomach fundus. The hiatal hernia defect was repaired with a constrictive primary suture. Roux-en-Y anastomosis was externally constricted, and the antidumping procedure was applied. There was no other procedure in the abdomen that could injure the bile ducts such as liver hilar and duodenal stump dissection. A penrose drain was placed under the left lobe of the liver at the level of the cardioesophageal junction. There was a 150 mL bile leak in 24 hours after the first day of surgery. Bile leakage was detected with MRCP on the left side of the left triangular ligament of the liver (Figures 1 and 2). The patient was reoperated, and no pathology was observed except for aberrant bile duct (a 3 mm diameter) with bile leakage at the site where the left liver triangular ligament (appendix fibrosa hepatis) was dissected at the observation by following the drain (Figure 3). The injured bile duct was ligated with the 3/0 prolene suture. On 7 day after the operation, all complaints resolved, and the patient was discharged without any problems in the postoperative period.

## 3. Discussion

The bile ducts do not have a constant structure, so the surgeons can have undesired surprise during the gastrointestinal system surgery. To avoid these situations, it is necessary to use imaging methods for diagnosis of the biliary tract before surgery, especially in women [2, 3, 5, 6]. It is somewhat more difficult to predict this situation in cases of previous gastric surgery, such as the one described by Iso et al. [6]. This is even more difficult, especially since it is not possible to endoscopically view the anatomy of the bile ducts during Billroth-2 gastrectomy. We have not been able to perform endoscopic imaging because of the presence of Billroth-2 gastrectomy and Roux-en-Y gastrojejunostomy. Today, however, this difficulty can be overcome by MRCP examination [1, 3, 6]. We also used MRCP in this case where endoscopic imaging could not be done, and we diagnosed bile leak with this method.

Conservative approaches for the management of bile leakages and the need for surgical intervention may vary according to the anatomical localization and the amount of the leakage and the clinical condition of the patient [3]. In cases where the bile ducts can be reached by endoscopic method, papillotomy and/or stenting can be used to reduce the intraluminal pressure of the bile ducts and reduce the leakage and stop them completely. However, the bile leak into peritoneum can cause peritonitis even when the drain is placed in the line or not [6]. In these cases, surgical intervention is inevitable. In our case, there was no bile peritonitis because the bile came directly to the drain, and daily diversion of the bile leak was approximately 150–200 mL. In this case, if we had the possibility of performing endoscopic sphincterotomy, we could follow this patient without performing open surgery. However, as mentioned above, the patient did not have a chance to do this operation because he had Billroth-2 gastrectomy and Roux-en-Y gastrojejunostomy. In addition, because of the abdominal surgery performed many times before, intensive intra-abdominal adhesions did not allow us to perform laparoscopic procedures and we had to do open surgery. During surgery, aberrant bile ducts and bile leak were observed at the localization of the left triangular ligament (appendix fibrosa hepatis) that was previously detected by MRCP. The leakage was sutured with prolene no. 3/0. Uysal and his colleagues reported that the variations of biliary tracts were seen more frequently in women than in men [1]. It was compatible in this situation that our case was also female. Extrahepatic and intrahepatic variations of the bile ducts are found more frequently on the right side, but the left-settling variations are less and the aberrant biliary tract in the left triangular ligament is an extremely rare case.

We consider that it is important to place a drain to the operation site for early controlling and treatment of bile leakage because of liver, biliary tract, pancreas, and upper gastrointestinal tract surgery, which are likely to encounter undesired surprises such as bile leakage due to the anatomical variations of the bile duct. With this idea, we recommend ligating this region if the left triangular ligament (appendix fibrosa hepatis) is dissected in surgery. In particular, it is necessary to keep in mind the possibility of aberrant bile ducts in the left triangular ligament in female patients with more biliary tract variations.

## References

[1] Uysal F., Obuz F., Uçar A., Seçil M., Igci E., Dicle O. (2014). Anatomic variations of the intrahepatic bile ducts: analysis of magnetic resonance cholangio-pancreatography in 1011 consecutive patients. *Digestion*.

[2] El Gharbawy R. M., Skandalakis L. J., Heffron T. G., Skandalakis J. E. (2011). Aberrant bile ducts, ‘remnant surface bile ducts,’ and peribiliary glands: descriptive anatomy, historical nomenclature, and surgical implications. *Clinical Anatomy*.

[3] Guan J., Zhang L., Chu J. P., Lin S. C., Li Z. P. (2015). Congenital left intrahepatic bile duct draining into gastric wall mimicking biliary reflux gastritis. *World Journal of Gastroenterology*.

[4] Kostov D. V., Kobakov G. L. (2011). Six rare biliary tract anatomic variations: implications for liver surgery. *Eurasian Journal of Medicine*.

[5] Shokouh-Amiri H., Fallahzadeh M. K., Abdehou S. T., Sugar M., Zibari G. B. (2014). Aberrant left main bile duct draining directly into the cystic duct or gallbladder: an unreported anatomical variation and cause of bile duct injury during laparoscopic cholecystectomy. *Journal of the Louisiana State Medical Society*.

[6] Iso Y., Kusaba I., Matsumata T. (1996). Postoperative bile peritonitis caused by division of an aberrant bile duct in the left triangular ligament of the liver. *American Journal of Gastroenterology*.

